# Epidemiology of Drowning in Bangladesh: An Update

**DOI:** 10.3390/ijerph14050488

**Published:** 2017-05-05

**Authors:** Aminur Rahman, Olakunle Alonge, Al-Amin Bhuiyan, Priyanka Agrawal, Shumona Sharmin Salam, Abu Talab, Qazi Sadeq-ur Rahman, Adnan A. Hyder

**Affiliations:** 1 Centre for Injury Prevention and Research, Bangladesh (CIPRB), House B162, Road 23, New DOHS, Mohakhali, Dhaka 1206, Bangladesh; al-amin@ciprb.org (A.-A.B.); abutalab01@ciprb.org (A.T.); 2 Johns Hopkins International Injury Research Unit, Department of International Health, Johns Hopkins Bloomberg School of Public Health, 615 N. Wolfe Street, Baltimore, MD 21205, USA; oalonge1@jhu.edu (O.A.); pagrawa6@jhu.edu (P.A.); ahyder1@jhu.edu (A.A.H.); 3 Centre for Child and Adolescent Health, icddr,b. 68 Shaheed Tajuddin Ahmed Sarani, Mohakhali, Dhaka 1212, Bangladesh; shumona@icddrb.org (S.S.S.); qsrahman@icddrb.org (Q.S.R.)

**Keywords:** drowning rate, fatal, non-fatal, rural areas, risk-factors, Bangladesh

## Abstract

Over one-quarter of deaths among 1–4 year-olds in Bangladesh were due to drowning in 2003, and the proportion increased to 42% in 2011. This study describes the current burden and risk factors for drowning across all demographics in rural Bangladesh. A household survey was carried out in 51 union parishads of rural Bangladesh between June and November 2013, covering 1.17 million individuals. Information on fatal and nonfatal drowning events was collected by face-to-face interviews using a structured questionnaire. Fatal and non-fatal drowning rates were 15.8/100,000/year and 318.4/100,000/6 months, respectively, for all age groups. The highest rates of fatal (121.5/100,000/year) and non-fatal (3057.7/100,000/6 months) drowning were observed among children 1 to 4 years of age. These children had higher rates of fatal (13 times) and non-fatal drowning (16 times) compared with infants. Males had slightly higher rates of both fatal and non-fatal drowning. Individuals with no education had 3 times higher rates of non-fatal drowning compared with those with high school or higher education. Non-fatal drowning rates increased significantly with decrease in socio-economic status (SES) quintiles, from the highest to the lowest. Drowning is a major public health issue in Bangladesh, and is now a major threat to child survival.

## 1. Introduction

The World Health Organization (WHO) estimated that 372,000 deaths occurred from drowning in 2012, which has made it the world’s third leading unintentional injury killer [1]. Over half of all drowning deaths occur among those under 25 years of age. Ninety-one percent of drowning deaths across all ages occur in low- and middle-income countries (LMICs) [2]. Fatal drowning rates among children in LMICs are 6 times higher than that of high-income countries (HICs), and several studies suggest that children aged 1–4 years are at the highest risk [3,4,5,6,7,8].

The Bangladesh Health and Injury Survey (BHIS) conducted in 2003–2004 revealed that drowning was the leading cause of deaths in children 1–17 years of age (28.6 per 100,000 children-years), and children 1–4 years-old were at highest risk (86.3 per 100,000 children-years) of drowning. The study also showed that the proportion of all deaths due to drowning among 1–4 year-olds was 26.0% in 2005; by 2011 this had increased to 42.0% [9,10].

In the HICs, drowning often occurs in recreational swimming pools [11,12,13], whereas in LMICs drowning happens in natural water bodies such as ponds, ditches, rivers, lakes, and dams [14,15,16]. Risk factors for childhood drowning in LMICs include, but are not limited to, inadequate supervision, male sex, lack of physical barriers between people and water bodies, and lack of swimming ability [8,16,17,18]. Lack of water safety awareness, risky behavior around water, and perceived risk are also considered important risk factors [19,20,21]. Travelling on overcrowded or poorly maintained vessels and water related disasters (e.g., flood, extreme rainfall, storm surges, and tsunamis or cyclones) are also common risk factors in all age groups globally [2].

Two supervisory tools, door barriers and playpens, were piloted in rural areas of Bangladesh, in an attempt to reduce drowning [17,18]. The findings suggested that both tools improved supervision; however, caregivers preferred playpens [18]. Another study explored the option of community crèches and reported that children who participated in the crèche program were 80% less likely to drown than those who did not participate [18,22].

However, as no nationwide childhood drowning prevention program has been implemented in Bangladesh, drowning continues to be the leading cause of death among children 1–4 years of age. Drowning also remains a leading cause of injury deaths among all age groups [23]. These estimates are based on modeled data and there is a lack of population-based data to describe the epidemiology, magnitude, and risk factors for drowning across all ages, and more specifically in children in Bangladesh. Such knowledge would be important for designing and implementing drowning prevention strategies that are responsive to the current risk factors not only in Bangladesh, but also in other LMICs with similar contexts.

The objective of this study was to describe the burden and risk factors of drowning for all demographics in rural Bangladesh, including children 1–4 years-old using data from a population-based census conducted in 2013. This paper aims to fill the gap in knowledge about the burden of drowning among all populations and provide updates on risk factors for drowning in rural Bangladesh.

## 2. Methods

A large-scale implementation project “Saving of Lives from Drowning” (SoLiD) was conducted in Bangladesh to test the effectiveness of childhood drowning prevention interventions. As part of the project, a baseline census was conducted between June and November of 2013 in 51 union parishads of seven rural sub-districts of Bangladesh: Matlab North, Matlab South, Daudkandi, Chandpur Sadar, Raiganj, Sherpur Sadar, and Manohardi. The census covered 1.17 million population and 270,387 households in these 51 union parishads.

Trained data collectors used pre-tested structured questionnaires to collect information from household heads or any adult above 18 years by face-to-face interviews. Data collection occurred in two stages. In the first stage, demographic, socio-economic, illness, and health-seeking information was collected for all members of the household. Household members who had any injury event were also identified during the first stage. In the second stage, information on injury morbidity and mortality were collected for both intentional and unintentional injuries. Injury was operationally defined as any external harm resulting from any assault, fall, cut, burn, animal bite, poisoning, transportation, operation of machinery, blunt objects, suffocation, or drowning related event resulting in the loss of one or more days of normal daily activities, school, or work. Drowning was described as the process of experiencing respiratory impairment from submersion or immersion in liquid [24]. Non-fatal drowning was operationally defined as survival from a drowning event. Information on all fatal injuries was collected over a one-year recall period, and over a six-month recall period for non-fatal injuries.

To ensure the quality of data, trained supervisors were recruited, and they observed 10% of interviews conducted by the data collectors, checked 10% of the collected data, and re-interviewed 2% of the households. In addition, field level research officers and managers were appointed to re-check all data for inconsistencies. If any inconsistency was found, the respective data collector was asked to revisit the household to collect correct information.

Ethical clearance for this study was obtained from the Institutional Review Boards of the Johns Hopkins Bloomberg School of Public Health in the USA, the Center for Injury Prevention Research, Bangladesh, and the International Centre for Diarrheal Disease Research, Bangladesh.

All records of fatal and non-fatal drowning were retrieved from the primary database for the current analysis. A description of the population by fatal and non-fatal drowning, sex, age, level of education, socio-economic status (SES) (computed based on a principal component analysis of household asset variables), and sub-districts was provided with proportion. Frequency distribution and proportion of different variables related to fatal and non-fatal drowning were also calculated. The descriptor variables included place of drowning, distance of water bodies from home, time of occurrence, and the seasonality of drowning. Drowning rates were calculated per 100,000 populations per year for fatal drowning and per 100,000 populations per 6 months for non-fatal drowning. These rates were further disaggregated by age, sex, SES, education, and sub-district levels. Fatal and non-fatal drowning outcomes were modeled as odds ratios comparing levels of independent variables such as age, sex, SES, and education using logistic regressions. Results from both bivariate and multivariate analyses are presented.

## 3. Results

The census covered 1.17 million people from seven selected sub-districts of Bangladesh. The proportion of females (51.5%) was slightly higher than males (48.5%). Among the total population, 9.6% were children under five years of age, 29.4% were 5 to 17 years of age, and about 61% were adults (18 years and over). Over one-quarter (25.3%) of the population had no formal education, however, about 60.0% had either primary or secondary level education. Considering the SES index, the population was divided into quintiles and the proportion of population in each category of SES index was almost the same, ranging between 18.1% and 21.6%. The proportion of the population in each sub-district varied depending upon the number of union parishads covered from the selected sub-district for the census (Table 1).

One hundred eighty-five fatal drowning cases in the year preceding the census and 3752 non-fatal drowning events in the preceding six months of the census were identified (Table 1). Among the non-fatal drowning cases, about 19.0% had multiple events. All cases of fatal drowning were unintentional in nature.

Fatal and non-fatal drowning rates were 15.8/100,000 per year and 318.4/100,000 per 6 months, respectively. Both fatal and non-fatal drowning rates were found higher among males (fatal: 19.0/100,000 per year; 95% confidence interval (95% CI) 15.7–23.1 and non-fatal: 372.6/100,000 per 6 months; 95% CI 357.1–388.8) than females (fatal: 12.8/100,000 per year; 95% CI 10.2–16.1 and non-fatal: 267.1/(254.3–280.5)). The difference of rates between male and female in non-fatal drowning was statistically significant.

The highest rates of fatal and non-fatal drowning were observed in children 1–4 years of age at 121.5/100,000 per year (95% CI 100.3–147.0) and 3057.7/100,000 in 6 months (95% CI 2948.0–3172.0), respectively. Among adults (18 years and over), the highest rate (8.4/100,000 per year; 95% CI 3.4–19.3) of fatal drowning was found among the 65 years and older age group; and the highest non-fatal drowning rates (28.4/100,000 per 6 months; 95% CI 24.1–33.49) were found among 25–64 year-olds.

Higher rates of fatal (12.5/100,000 per year; 95% CI 8.9–17.5) and non-fatal drowning (139.6/100,000 per 6 months; 95% CI 126.4–153.6) were observed among those who did not have any education compared to the educated groups.

The highest rates for both fatal (21.7/100,000 per year; 16.1–29.3) and non-fatal drowning (504.1/100,000; 474.7–535.3) were observed in the most deprived SES quintile, and with the increase of SES index the incidence rates decreased. However, the fatal drowning incidence rate was found to be slightly higher (13.0/100,000 per year) in the wealthiest quintile (highest) than the wealthy quintile (high) (11.3/100,000 per year).

Fatal drowning rates were found to be similar in all sub-districts, ranging from 16.7 to 20.3/100,000 per year, except in Sherpur Sadar (12.3/100,000 population per year) and Manohardi (12.2/100,000 population per year). The highest fatal drowning rate was observed in Chandpur Sadar (20.3/100,000 population per year) and the lowest in Manohardi (Table 2). Significant variations were found in comparisons of non-fatal drowning rates between sub-districts (Table 2), with the highest non-fatal drowning rates in Raiganj, followed by Daudkandi, Matlab South, and Matlab North.

Multiple logistic regression analysis showed that males were at higher risk of both fatal and non-fatal drowning than females. Children 1–4 years of age were 13.3 times (CI 3.3–54.0; *p* = 0.000) and 15.9 times (CI 11.2–22.5; *p* = 0.000) higher at risk of fatal and non-fatal drowning, respectively, than infants (<1 year). Although individuals in other older age groups were also at higher risk of fatal drowning, the odds ratios were not statistically significant. In the case of non-fatal drowning, the analysis showed a statistically significant higher risk in all age groups compared to infants. Individuals with no education had 3.7 times (CI 0.8–16.7; *p* = 0.1) and about 2.9 times (CI 1.3–6.7; *p* = 0.013) higher risk of fatal and non-fatal drowning, respectively, than those who had secondary level education or higher. With the decrease of SES quintile from the highest to the lowest, the risk of fatal and non-fatal drowning increased; this association was, however, only significant for non-fatal drowning events (Table 3).

**Figure 1:**
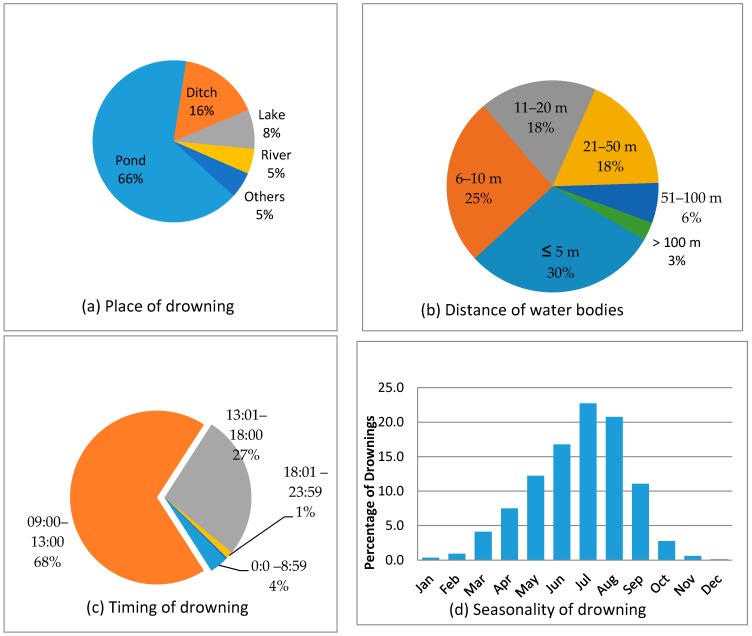
Factors associated with fatal and non-fatal drowning: (**a**) Place of drowning; (**b**) Distance of water bodies; (**c**) Timing of drowning; (**d**) Seasonality of drowning.

Natural bodies of water such as ponds, ditches, lakes, and rivers were common places of drowning. Ponds were the most common place (66.0%) of drowning in Bangladesh. About three-quarters (73.0%) of drowning took place in bodies of water within 20 m from the victims’ house. Almost all (95.0%) drowning occurred during the daylight between 0900 h and 1800 h, of which almost two-thirds occurred before 1300 h. The study revealed a seasonal pattern of drowning which showed an increase of drowning during monsoon, with peaks in July and the winters (November–January) relatively free of drowning events (Figure 1).

## 4. Discussion

As in other LMICs, the reporting system for deaths and health related events is weak in Bangladesh. Thus, it is very difficult to ascertain the burden of diseases and injuries in the country based on routinely collected data. In addition, most recent population-based research on the burden of drowning in the country is quite dated [25,26,27,28]. Therefore, this study, using recent population-based data, provides a comprehensive update on the burden and epidemiology of drowning in Bangladesh. In this survey, household visits were conducted to collect relevant data on fatal and non-fatal drowning events for over 1 million people.

This study suggests that across all ages, the fatal drowning rate was 15.8 per 100,000 people per year and the non-fatal drowning rate was 318.4 per 100,000 per 6 months in rural Bangladesh. According to the WHO Global Report on Drowning, rates of drowning in LMICs, such as Bangladesh, are higher than in HICs, and in comparing LMICs, the fatal drowning rate in Bangladesh is 2 to 5 times higher than rates from most other LMICs based on the 2012 WHO Global Health Estimates [1,2]. These findings suggest that the burden of global drowning may be disproportionately borne by a few LMICs, including Bangladesh; thus, initial efforts on global drowning prevention may focus on such countries.

Within Bangladesh, while no variations were noticed in the fatal drowning rates, significant variations were observed in non-fatal drowning rates comparing geographical regions in the seven Upazilas surveyed in rural Bangladesh, and sub-districts such as Raiganj, Daudkandi, Matlab North and Matlab South had significantly higher rates. These differences in the non-fatal drowning relative to drowning rates may be indicative of the differences in the level of awareness about the risk of drowning, exposure to water bodies, and coverage of drowning prevention activities between these sub-districts. For instance, Raiganj was seen to have the highest fatal and non-fatal drowning events, in spite of having SwimSafe programs and crèches in their communities for over a decade [26]. It is probable that due to awareness among residents in the community about injury prevention programs, there were higher self-reporting instances. In addition, Matlab North, Matlab South, Chandpur Sadar, and Daudkandi have more natural water bodies in and around them, which when considered along with all other risk factors, puts its residents at a higher risk of drowning events.

The rates for both fatal and non-fatal drowning were higher among males when compared to females, which is similar to other LMICs, as reported by the WHO Global Report on Drowning [2]. The higher risk among males can probably be attributed to higher exposure to risky situations, increased risk taking behavior, and involvement in activities outside the home among males [27].

The highest rates of fatal and non-fatal drowning were observed in children 1–4 years of age followed by 5–9 year-olds children. These findings are similar to findings from a previously conducted national survey, Bangladesh Health and Injury Survey (BHIS) 2005, and reflect the trend that rural children aged 1–4 years are the worst affected by drowning in Bangladesh, and that this trend has not changed over the past decade [2,8]. Similar findings have also been reported in other East-Asian countries such as Thailand, Vietnam, and China [28], and countries in the Western Pacific Region [29]. The increased risk of drowning among children under five years of age has been associated with lack of adequate adult supervision, combined with environmental risks, and behavioral factors such as increased curiosity among toddlers, lack of ample dexterity and co-ordination, and limited cognitive awareness of their surroundings [2,15,26].

Low educational level and poor socio-economic status were associated with higher drowning rates for both fatal and non-fatal events, and similar findings have also been reported in previous studies from Bangladesh [30,31,32]. Such disparities in drowning outcomes may suggest disproportionate access to safe environments, knowledge around drowning prevention, and relevant rescue and health services when comparing populations of different socioeconomic status. Thus, these results highlight the need for prioritizing populations from low socioeconomic status backgrounds in drowning prevention efforts.

In this study, most drowning events occurred in ponds and ditches, almost all located within 20 m from the residences of victims, and most frequently during the annual monsoon season, similar to findings reported in prior studies [15,22,28]. Given the tropical climate in Bangladesh, monsoon rainfalls may lead to floods, and increased water levels in rivers, canals, ponds, and ditches, which puts children and other individuals at higher risk of drowning [33]. Virtually all (95%) drowning events occurred in the daylight hours. This pattern of seasonality and time of drowning are in accordance with other studies [25,28,33,34].

A major limitation of this study is in its definition of near-drowning events. There is no standard operational definition of non-fatal drowning. In the previous 2005 BHIS survey, a non-fatal drowning event was defined as a victim who sought medical care or had at least a three-day work loss or absence from school or inability to do normal daily activities, but in the current study, it was defined just as survival from a drowning event associated with care-seeking and or at least one-day work loss or absence from school in the current study. Hence, findings on non-fatal drowning events of this study are not comparable to the BHIS study. Notwithstanding this limitation, while the absolute rates of non-fatal drowning among children less than 18 years of age in this study were different from a previous study, the drowning pattern (higher rates among this age group) was found to be similar [9].

Additionally, the study covered predominantly rural areas of Bangladesh; thus, the findings may not be generalizable to urban areas of Bangladesh. However, as the topography of rural Bangladesh is homogeneous in nature, the study findings are generalizable to other rural areas of Bangladesh.

## 5. Conclusions

The study suggests that the magnitude of fatal and non-fatal drowning is very high in rural communities of Bangladesh, and that drowning rates may be on the rise. Male gender, children under 5 years of age, having limited to no education, and being of lower socio-economic status were associated with increased risk of fatal and non-fatal drowning events. It is evident that drowning is a neglected public health problem in Bangladesh and that the child population is the most vulnerable. Evidence based interventions such as playpens and community crèches are effective in preventing drowning mortality in children under five years of age. It is expected that the consideration and implementation of the research findings from SoLiD will provide a wealth of knowledge in preventing child drowning. There is also a need for a national effort for drowning prevention for all ages; such an effort could incorporate other age-specific drowning prevention strategies to reduce the overall burden of drowning in rural Bangladesh.

## Figures and Tables

**Table 1:** Description of population by sex, age, level of education, socio-economic status (SES) index, sub-districts, and fatal and non-fatal drowning outcomes.

Characteristics	Counts (N)	Frequency (%)
**Sex**		
Male	567,674	48.54
Female	601,919	51.46
**Age Group**		
<1 year	22,141	1.89
1–4 years	90,523	7.74
5–9 years	139,728	11.95
10–14 years	142,121	12.15
15–17 years	62,098	5.31
18–24 years	133,534	11.42
25–64 years	508,059	43.44
65+ years	71,389	6.10
**Level of Education**		
No education	295,314	25.3
Primary	407,923	34.9
Secondary	289,658	24.8
A levels and above	63,873	5.5
Not applicable (<5 years)	112,664	9.6
**Socio-Economic Index**		
Lowest	211,610	18.1
Low	218,695	18.7
Middle	238,371	20.4
High	247,716	21.2
Highest	253,210	21.6
**Sub-Districts**		
Matlab North	265,897	22.7
Matlab South	209,772	17.9
Chandpur Sadar	128,356	11.0
Raiganj	104,357	8.9
Sherpur Sadar	228,519	19.5
Manohardi	204,319	17.5
Daudkandi	28,373	2.4
**Drowning**		
Fatal (1 year recall)	185	0.016
Non-fatal (6 months recall)	3752	0.321

**Table 2:** Fatal and non-fatal drowning rates (per 100,000) by sex, age, level of education, SES index, and sub-districts.

Variables	Fatal Drowning		Non-Fatal Drowning	
	Population (N)	Rate/100,000/Year (95% CI)	Population (N)	Rate/100,000/6 Months (95% CI)
**Sex**				
Both	1,169,593	15.8 (13.6–18.3)	1,178,256	318.4 (308.4–328.8)
Male	567,674	19.0 (15.7–23.1)	573,225	372.6 (357.1–388.8)
Female	601,919	12.8 (10.2–16.1)	605,031	267.1 (254.3–280.5)
**Age in Years**				
<1 year	22,141	9.0 (1.6–36.4)	21,594	171.3 (122.4–234.7)
1–4 years	90,523	121.5 (100.3–147.0)	91,737	3057.7 (2948.0–3172.0)
5–9 years	139,728	22.9 (15.9–32.7)	141,024	465.9 (428.3–499.6)
10–14 years	142,121	5.6 (2.6–11.6)	143,206	37.0 (28.0–48.8)
15–17 years	62,098	3.2 (0.6–13.0)	62,580	14.4 (7.0–28.38)
18–24 years	133,534	6.7 (3.3–13.3)	134,535	19.3 (12.9–28.76)
25–64 years	508,059	3.1 (1.9–5.2)	514,264	28.4 (24.1–33.49)
65+ years	71,389	8.4 (3.4–19.3)	69,316	27.4 (17.0–43.69)
**Level of Education**				
No education	295,314	12.5 (8.9–17.5)	296,357	139.6 (126.4–153.6)
Primary	407,923	6.9 (4.6–10.1)	412,140	108.8 (98.1–118.3)
Secondary	289,658	1.7 (0.6–4.3)	292,118	15.9 (11.7–21.2)
Higher secondary level and above	63,873	3.1 (0.5–12.6)	64,158	10.9 (4.8–23.6)
Not applicable (<5 children)	112,664	100.3 (83.0–121.0)	113,331	2522.5 (2418.0–2601.0)
**SES Index**				
Lowest	211,601	21.7 (16.1–29.3)	213,242	504.1 (474.7–535.3)
Low	218,695	18.7 (13.6–25.7)	220,666	375.2 (350.3–401.8)
Middle	238,371	15.5 (11.1–21.6)	240,313	297.5 (276.3–320.3)
High	247,716	11.3 (7.7–16.6)	249,546	253.3 (234.1–274.0)
Highest	253,210	13.0 (9.1–18.5)	254,489	197.3 (180.6–215.5)
**Sub-District**				
Matlab North	265,897	17.3 (12.8–23.3)	267,748	306.5 (284.0–326.2)
Matlab South	209,772	16.7 (11.8–23.5)	213,691	531.5 (491.9–553.5)
Chandpur Sadar	128,356	20.3 (13.5–30.1)	128,671	194.0 (170.6–219.5)
Raiganj	104,357	19.2 (12.0–30.2)	106,044	885.4 (816.7–929.6)
Sherpur Sadar	228,519	12.3 (8.3–18.0)	228,591	107.6 (94.78–122.2)
Manohardi	204,319	12.2 (8.1–18.4)	204,551	113.5 (99.51–129.2)
Daudkandi	28,373	17.6 (6.5–43.7)	28,960	602.7 (507.0–687.3)

CI: Confidence Interval.

**Table 3:** Association between socio-demographic factors and drowning events, fatal and non-fatal.

Characteristics	Fatal Drowning				Non-Fatal Drowning			
	OR (95% CI) Unadjusted	*p* Value	OR (95% CI) Adjusted	*p* Value	OR (95% CI) Unadjusted	*p* Value	OR (95% CI) Adjusted	*p* Value
**Sex**								
Male	1.5 (1.1–2.0)	0.008	1.4 (1.0–1.9)	0.030	1.4 (1.3–1.5)	0.000	1.2 (1.1–1.3)	0.000
Female	1		1		1		1	
**Age Groups (Years)**								
<1 year	1		1		1		1	
1–4 years	13.5 (3.3–54.5)	0.000	13.3 (3.3–54.0)	0.000	18.4 (13.3–25.4)	0.000	15.9 (11.2–22.5)	0.000
5–9 years	2.5 (0.6–10.6)	0.20	1.3 (0.2–10.1)	0.818	2.7 (2.0–3.8)	0.000	1.5 (0.6–3.6)	0.423
10–14 years	0.6 (0.1–2.9)	0.56	0.5 (0.1–4.4)	0.537	0.2 (0.1–0.3)	0.000	0.2 (0.1–0.4)	0.000
15–17 years	0.4 (0.1–2.5)	0.30	0.4 (0.0–4.2)	0.428	0.1 (0.04–0.17)	0.000	0.1 (0.0–0.2)	0.000
18–24 years	0.8 (0.2–3.5)	0.71	0.7 (0.1–4.7)	0.673	0.11 (0.07–0.19)	0.000	0.1 (0.0–0.2)	0.000
25–64 years	0.4 (0.1–1.5)	0.16	0.2 (0.0–1.3)	0.084	0.67 (0.12–0.24)	0.000	0.1 (0.0–0.2)	0.000
65+ years	0.9 (0.2–4.6)	0.93	0.3 (0.0–3.0)	0.323	0.16 (0.09–0.28)	0.000	0.1 (0.0–0.2)	0.000
**Level of Education**								
No education	4.0 (1.0–16.6)	0.05	3.7 (0.8–16.7)	0.100	12.8 (6.1–27.0)	0.000	2.9 (1.3–6.7)	0.013
Primary	2.2 (0.5–9.2)	0.28	1.3 (0.3–6.1)	0.728	9.9 (4.7–20.9)	0.000	1.4 (0.6–3.1)	0.480
Secondary	0.6 (0.1–2.8)	0.47	0.6 (0.1–3.0)	0.506	1.4 (0.7–3.2)	0.37	1.1 (0.5–2.7)	0.776
A levels and above	1		1		1		1	
**SES Index**								
Lowest	1.7 (1.1–2.6)	0.02	1.3 (0.8–2.1)	0.234	2.6 (2.3–2.9)	0.000	2.0 (1.8–2.3)	0.000
Low	1.4 (0.9–2.3)	0.12	1.3 (0.8–2.1)	0.250	1.9 (1.7–2.1)	0.000	1.8 (1.6–2.1)	0.000
Middle	1.2 (0.7–1.9)	0.46	1.1 (0.7–1.8)	0.656	1.5 (1.4–1.7)	0.000	1.4 (1.3–1.6)	0.000
High	0.9 (0.5–1.4)	0.57	0.8 (0.5–1.4)	0.500	1.3 (1.1–1.5)	0.000	1.4 (1.2–1.6)	0.000
Highest	1		1		1		1	

OR: Odds Ratio.
